# Medical Association of Central New York

**Published:** 1897-02

**Authors:** 


### Society Proceedings.

### MEDICAL ASSOCIATION OF CENTRAL NEW YORK.

THE twenty-ninth annual meeting of the Medical Association of Central New York was held at the Chamber of Commerce, Rochester, N. Y., Tuesday, October 20, 1896.

The association was called to order at 11 o'clock by the president, Dr. W. S. CHEESMAN, of Auburn. The reading of the minutes of the last meeting was dispensed with and, on motion of the Secretary, were approved as printed in the proceedings of last year.

The president then announced the following committees :

*Credentials*—Drs. C. W. Wilbor, Monroe ; John Gerin, Cayuga ; H. L. Chace, Wayne.

*Business*—Drs. A. L. Benedict, Erie ; H. B. Wright, Onondaga; B. W. Loomis, Onondaga.

*Reception*—Drs. E. B. Angell, J. O. Roe, H. T. Williams, T. O. Tait and W. B. Jones, Monroe.

*Ethics*—Drs. Clark, Onondaga ; B. C. Loveland, Ontario, and D. R. Burrell, Ontario.

*Publication*—Drs. Wm. C. Krauss, Erie ; C. A. Van der Beek, Monroe ; S. L. Elsner, Onondaga.

The report of the treasurer was read and showed a balance on hand of $116.77.

The report was accepted and ordered placed on file.

The chairman of the Committee on Credentials announced the following list of delegates who had complied with the requirements of the by-laws : John O. Palmer and Susan J. Otis, Cayuga ; W. M. Brown, Benjamin Wilson, Anna Craig, E. A. Cady-Harris, Evelyn Baldwin, F. W. Maloney and Frank F. Dow, Monroe ; B. W. Loomis, Geo. Edward Clark, I. Harris Levy, Juliet E. Hanshett, H. B. Wright, Onondaga ; S. R. Wheeler, Ontario ; J. W. Eddy, Oswego, and L. H. Smith, of Wayne, and, on motion of the secretary, they were elected to permanent membership.

The following members and delegates registered :

| Name. | County. | Name. | County. |
|---|---|---|---|
| A. L. Benedict, | Erie. | F. W. Higgins, | Cortland. |
| S. R. Wheeler, | Ontario. | L. H. Smith, | Wayne. |
| B. C. Loveland, | Ontario. | M. R. Caresly, | Orleans. |

Name. County.

John Gerin, Cayuga.
E. S. Foreman, Cayuga.
W. D. Wolff, Monroe.
H. L. Chace, Wayne.
J. C. Davis, Monroe.
Chas. E. Doubleday, Yates.
F. H. Stephenson, Onondaga.
H. B. Wright, Onondaga.
R. Cook, Monroe.
C. H. Richmond, Livingston.
J. A. Richty, Ontario.
I. Harris Levy, Onondaga.
A. B. Randall, Onondaga.
J. Henry Dowd, Erie.
Alfred Mercer, Onondaga.
B. W. Loomis, Onondaga.
L. Howe, Erie.
G. E. Clark, Onondaga.
Geo. G. Jones, Livingston.
W. T. Linn, Madison.
E. H. Gray, Monroe.
H. L. Elsner, Onondaga.
C. O. Jackson, Ontario.
John O. Palmer, Cayuga.
Wm. M. Brown, Monroe.
F. S. Snow, Onondaga.
M. A. Brownell, Wayne.
Nathan Jacobson, Onondaga.
Allen Cone, Onondaga.
Susan G. Otis, Cayuga.
J. P. Creveling, Cayuga.
M. S. Collier, Monroe.
T. H. Hallett, Wayne.
J. A. Robson, Ontario.
J. F. Myers, Onondaga.
A. F. Sheldon, Wayne.
E. B. Angell, Monroe.
I. E. Harris, Monroe.
F. B. Storer, Orleans.
W. S. Ely, Monroe.

Name. County.

D. M. Totman, Onondaga.
W. Rider, Monroe.
J. W. M. Cauley, Monroe.
T. Oliver Tait, Monroe.
Thos. Jameson, Monroe.
L. Allen Walker, Monroe.
C. A. Van der Beek, Monroe.
Frederick Sefton, Cayuga.
F. D. Putnam, Cayuga.
E. H. Howard, Monroe.
L. B. Andrews, Wayne.
W. V. Ewers, Monroe.
A. L. Beahan, Ontario.
O. J. Hallenbeck, Ontario.
W. E. King, Wyoming.
E. Cady-Harris, Monroe.
M. R. Carson, Ontario.
L. W. Horst, Monroe.
F. Kenyon, Cayuga.
A. B. Straight, Wyoming.
A. R. Gumberts, Monroe.
W. L. Conklin, Monroe.
A. M. Mead, Ontario.
E. H. Earl, Monroe.
Benj. Wilson, Monroe.
E. L. Mooney, Onondaga.
W. S. Hare, Monroe.
J. E. Ottaway, Monroe.
H. R. Nettleton, Monroe.
C. H. Towlerton, Wayne.
John Zimmer, Monroe.
C. T. La Moure, Monroe.
E. B. Potter, Monroe.
Marion Craig Potter, Monroe.
Anna Craig, Monroe.
F. W. Zimmer, Monroe.
L. K. Mazger, Monroe.
Thos. Mooney, Monroe.
P. C. Guinan, Monroe.
W. J. Herriman, Monroe.

| Name. County. | Name. County. |
|---|---|
| Lewis W. Rose, Monroe. | Le Roy L. Hollis, Oswego. |
| B. S. Coleman, Monroe. | J. E. Hanchett, Onondaga. |
| R. T. French, Monroe. | R. S. Carr, Wayne. |
| E. E. French, Monroe. | J. H. Finnessy, Monroe. |
| M. J. Slaight, Monroe. | F. A. Jones, Monroe. |
| Evelyn Baldwin, Monroe. | L. H. Woodruff, Monroe. |
| Alvin A. Hubbell, Erie. | J. M. Ingersoll, Monroe, |
| Lorenzo Barrows, Erie. | W. W. Williams, Monroe. |
| L. T. Gandy, Monroe. | Willard D. Becker, Monroe. |
| A. H. Thomas, Monroe. | H. C. Phillips, Monroe. |
| Cornelia White Thomas, Monroe. | F. W. Spaulding, Ontario. |
| A. P. Maine, Monroe. | J. D. Briggs, Wayne. |
| F. W. Maloney, Monroe. | D. G. Mason, Monroe. |
| Chas. Reitz, Monroe. | L. A. Weigel, Monroe. |
| Jos. W. Eddy, Oswego. | F. H. Lawers, Monroe. |
| C. R. Barber, Monroe. | Wm. C. Krauss, Erie. |
| F. D. Andrew, Monroe. | D. F. Curtis, Monroe. |
| W. B. Jones, Monroe. | Thomas A. O'Hare, Monroe. |
| P. D. Carpenter, Monroe. | A. C. W. Graham, Monroe. |
| R. V. Bamber, Orleans. | Jno. H. Pratt, Ontario. |
| Fred Gould, Orleans. | J. F. Sherman, Monroe. |
| Alonzo Redman, Monroe. | G. E. H. Jones, Livingston. |
| J. W. Flick, Monroe. | Archibald Dann, Monroe. |
| S. Hayward, Monroe. | W. J. Howe, Monroe. |
| G. Theo. Fischer, Monroe. | E. R. Hardenbrook, Monroe. |
| E. H. Lapp, Monroe. | F. East, Monroe. |
| B. F. McDowell, Ontario. | H. P. Sheldon, Monroe. |
| Peter Stockschlader, Monroe. | O. E. Jones, Monroe. |
| J. D. Dunning, Monroe. | Ida M. Porter, Monroe. |
| W. J. Hennessy, Wayne. | S. L. Elsner, Monroe. |
| Frank F. Dow, Monroe. | C. A. Huber, Monroe. |
| H. T. Williams, Monroe. | R. M. Moore, Monroe. |
| John O. Roe, Monroe. | Nathan W. Soble, Monroe. |
| C. C. Thayer, Ontario. | Henry H. Corell, Monroe. |
| Wm. S. Cheesman, Cayuga. | F. C. Curtis, Albany. |
| J. E. Weaver, Monroe. | |

The president, Dr. W. S. Cheesman, of Auburn, N. Y., delivered the annual address, entitled Back to Nature.

Upon motion a vote of thanks was extended by the association to the president for his excellent address.

The chairman of the business committee announced that papers would be read in the order in which they appeared in the secretary's program, and requested that each reader confine himself to twenty minutes.

Papers were then read as follows: (1) Should Credé's method be made obligatory in public institutions? By Lucien Howe, M. D., Buffalo, N. Y. (2) Ether anesthesia—its semicentennial anniversary. By Nathan Jacobson, M. D., Syracuse, N. Y.

*Discussion:*

Dr. E. M. Moore, Sr., of Rochester: I have several little things to say. I strongly suspect that I am the oldest man in the profession here and can tell you of the practice in the old days. Now, in those days, the function of the surgeon was a very serious one. There was an amount of skill required which is not called for at the present time; an extra skill we can scarcely conceive of, now that anesthesia has rendered fifty men competent, where only men of great skill could be surgeons in the early days. It is all very well to tell what used to be done; how patients were held down on the table; what an extraordinary annoyance it was to have the women helpers in the room and what a tragic scene it was.

In this admirable paper of Dr. Jacobson's he has given us a good description of many things, but as regards that of the government support, that would be a very dangerous precedent. Any such support applies to other professions in life also.

Milton sold Paradise Lost for five pounds; and this is the fate of higher mankind, which has always persecuted its great benefactors. Christ was crucified. Socrates was made to swallow the Hemlock. Remember the old distich:

> Seven cities claimed Homer dead,
> Through which the living Homer begged his bread.

Dr. William S. Ely, of Rochester: I have listened with interest to this valuable paper. Having just read a full report of the addresses at the semicentennial celebration of the discovery of ether in Boston, I wondered what Dr. Jacobson would find in the preparation of his paper that had not already been published; but, after hearing his brilliant article, I am prepared to say that Boston should have sent to Syracuse for her chief orator on the occasion of this ether anniversary.

(3) Hysteria. By B. C. Loveland, M. D., Clifton Springs, N. Y.

*Discussion :*

Dr. E. B. ANGELL, of Rochester : The subject of hysteria is too broad a one for brief discussion. Of its pathology we have little exact knowledge. In its expression it simulates almost every known disorder. It is said, indeed, that no sooner has the symptomatology of a new disease been established than we meet with its hysterical counterfeit. Recently in a journal of neurology I encountered an article on The hysterical forms of Raynaud's disease, which well illustrates the force of this relationship.

Unquestionably hysteria is a psychical disorder and more or less allied to a perversion of the subconscious activity, of which hypnosis is a kindred manifestation. Furthermore, it is one of the marked stigmata of degeneration. There is a decided exaltation of the *ego* with a corresponding decrease in the control nominally exercised by the higher intellectual centers. The individual is no longer stable, but the prey of every excessive, ingoing sensory impression, whether it be exalted physical sensation or undue mental emotion.

If we accept this view as a basis of rational treatment, the prime importance of securing healthy physical action as a basis for the reëstablishment of nominal self-control becomes apparent. Succeeding this, mental and moral education are scarcely secondary in importance, to secure which oftentimes seclusion from the hurtful sympathy of friends is absolutely necessary. Hysterical manifestations are not imaginary, but real, and demand the utmost ingenuity and patience of the physician for their removal.

(4) Breathing and bicycling : the ill effects of mouth-breathing upon the throat and lungs and the importance of free nasal respiration in the exercise of wheeling. By John O. Roe, M. D., Rochester.

*Discussion :*

Dr. SARGENT F. SNOW, of Syracuse : The paper just read is full of good points, and I have no doubt that the observations regarding bicycle riders are correct.

There are many here who do more or less nasal work, and we all know that one of the worst obstacles in our way is the recurrence in a year or two of some membranous thickenings, even though we did our work thoroughly and well. Experience teaches me that nearly all patients who do not get good results from surgical work are still clinging to their pernicious habit of mouth-breathing. In fact, this habit in some cases is most persistent, and unless broken up will tend to bring nasal surgery into disrepute.

Dr. Roe's remarks likening the nasal passages to a roadway grown up with weeds is a very good one. It is surely a fact ; and further, if we use our mouth for breathing purposes the nasal, post-nasal, aural and laryngeal membranes will become very sensitive and affected by chronic inflammation.

I think that Dr. Roe's paper is one that all here who do nose, throat and ear work can most heartily endorse.

Dr. William S. Ely, of Rochester : I desire to express my approval of the views advanced by Dr. Roe in his excellent article. I do not know that I fully share the views expressed by throat specialists as to the dangers of mouth-breathing. I have looked upon the mouth as a supplemental breathing organ, constantly called upon in conversation and in all forms of violent exercise.

The demand for an increased amount of oxygen that is felt in active exercise cannot always be met by the nostrils, even when they are unobstructed, and I think there are times when we all feel thankful that we can open our mouths for supplemental breathing. In a pure country, sea or mountain air, I relish drinking it in as much as some persons enjoy a glass of champagne.

It seems to me that there are, therefore, some qualifications to the advice, "keep your mouth shut when breathing." I want to say, however, that the nares should be kept free and unobstructed and that Dr. Roe is correct in all that he has said on that point.

Dr. B. W. Loomis, of Syracuse : I have been much interested in the paper read by Dr. Roe and being a bicycle rider myself, of several years' standing, I have a particular interest in the points he raises. From a physiological point of view, the paper is an admirable one. We all admit the claim that the nose is the channel for breathing and that riders of "the wheel," as well as others, should keep the mouth shut and do the greater part of their breathing through the nose. This position is, at the present time, unassailable.

I think everyone will agree, also, that if bicycle riders breathe too much through the mouth, or fail to use the nasal passages for the purpose for which they were intended, why, then, the pastime or exercise is likely to be injurious.

But the question of importance to me and to riders generally is: Do bicycle riders as a rule breathe in this injurious way? I would like to ask Dr. Roe to come directly to the point in the paper, as to whether it has been found that riders are suffering more from throat trouble, from nasal or other troubles of a like nature, than they did before they rode the wheel. Does the practice of "wheeling" develop any great liability to these difficulties?

As bearing upon this question and serving as a basis for the opinion I have partially formed, I may say that I am a member of one of the largest athletic organisations in the state—the Syracuse Athletic Association. This association numbers between 600 and 700 members, a majority of whom are riders of bicycles. I have ridden a wheel regularly during the past four years and have been in general practice for nearly twenty years, so it seems that I am in a position to discover whether bicycle riders are apt to develop these particular ailments. In view of the fact that most patients, when they first discover any local trouble, usually consult the general practitioner before going to the specialist, it seems to me that I should have had my attention called to any increase of these ailments among riders. As a matter of fact, I have been unable to see that wheelmen are especially likely to contract throat or nasal troubles as a result of riding and I do not think there is anything in the exercise tending to produce these troubles.

Dr. Roe: In closing the discussion I have but a few words to say. The subject of the paper was suggested entirely by the frequency with which patients came under my observation suffering from affections of the throat and which had been much aggravated by the increased amount of mouth-breathing, while riding their wheels, necessitated by the obstructed condition of their nasal passages. In reply to Dr. Loomis' question, Does bicycle riding tend to develop a liability to affections of the throat? I would say that it certainly does not, so long as the person is able to breathe freely through his nose. If, on the other hand, he is obliged to breathe through his mouth for any length of time, the increased amount of air respired during the active exercise of wheeling tends directly to aggravate any chronic affection of the throat, or it may induce affections of the throat which had not previously existed, as was pointed out in the paper.

I fully concur with all that Dr. Snow has said, but am surprised that Dr. Ely should regard nasal respiration as of so slight importance. It is fortunate, of course, that we have the mouth to utilise for supplementary respiration. Many would suffer very seriously if they were obliged to get on with the small amount of air which they are able to obtain through their obstructed nasal passages. During ordinary conversation, more or less air is taken in through the mouth without special detriment, as I have stated ; but it is the continuous respiration through the mouth which produces serious results. These ill-effects are not only observed during wheeling, when continued for a length of time, but are also seen in persons who sleep with the mouth wide open, taking all or nearly all of their air through the mouth. Not alone in the throat are these consequences of mouth-breathing observed, but also in the distorted countenance. The influence of mouth-breathing upon the physiognomy was first pointed out by Catlin and depicted in a number of striking illustrations.

The more the nose is studied, the more we recognise the importance of nasal respiration and the protection which it gives to the organs below. It is a fact of daily observation that no person can breathe continuously through his mouth without inducing more or less disturbance in the respiratory organs below, and this disturbance steadily increases in proportion to the length of time that the habit is indulged in.

(6) Friedreich's ataxia. By Dr. F. H. Stephenson, Syracuse.

The meeting was then adjourned until 2 o'clock P. M., and luncheon was served in the Chamber of Commerce, through the courtesy of the Monroe County Medical Society. The association reassembled at 2.30 P. M., at which time the reading of papers was continued.

(7) The use of the stomach-tube. By Dr. A. L. Benedict, Buffalo.

*Discussion :*

Dr. B. W. Loomis, of Syracuse : Regarding the principles involved in the uses of stomach-tubes, Dr. Benedict has covered the ground thoroughly in his paper. There are, however, some matters of a mechanical nature in connection with the subject to which I should like to call attention. To begin with, the introduction of a tube and washing out the stomach is a problem, or rather a series of problems, in hydraulics. On this account the features of the operation are entitled to our consideration. The outer, or funnel-end of the stomach-tube is usually of soft rubber, and will hold when filled with water about five ounces. It is so soft and flabby that in practice it is difficult to handle. I have found much satisfaction in using a large aluminum funnel, which is thrust into the rubber tube after the funnel-end is cut off. This aluminum funnel is light and is provided with a handle, making it easier to hold, and it will hold when full nearly a quart of water. The weight and pressure of this volume of water will produce a more thorough lavage. It loosens mucus and particles of food to a greater degree, and is, therefore, apt to insure a complete emptying of the organ.

Another feature in the mechanical problem is the relation of the inside diameter or bore of the tube to the outside diameter. On making a tour of inspection among the drug stores, the dealers in surgical instruments and rubber goods, and comparing different tubes kept in stock, I learned that there is considerable difference in tubes as regards their internal diameter. It is obvious that the efficiency of lavage depends to some extent on the internal diameter of the rubber tube. It should be as large as possible consistent with strength and stiff enough not to collapse. Supposing we have a choice between two tubes of equal diameter externally, it is plain that the one having the largest bore or internal diameter will permit the passage of water more freely and, what is more important, will allow larger particles of undigested food and débris to come forth from the stomach that could not pass through a tube of smaller diameter. With a view of determining experimentally the efficiency of different tubes of the same size outwardly, I have connected them with a large bottle and by syphonage have found that some of those offered for sale are so small that it requires twice as long to empty the bottle as with other tubes of the same external diameter, but having a larger bore. It follows, as a matter of course, that such a tube would not efficiently remove large pieces of undigested food, and even mucus will not engage and pass through the narrow orifice.

Another reason for using tubes of as large caliber as possible is found in the fact that with some patients it is desirable to accomplish the work in as short space of time as may be, because these patients will almost invariably vomit and expel the tube after a moderate lavage.

(8). Sciatica—A neuritis, rarely a neuralgia. Illustrative cases. By Dr. E. B. Angell, Rochester.

*Discussion:*

Dr. J. W. EDDY, of Oswego: I wish to say one thing of the chloride of methyl, C. $H_3$. Cl. which I have used. Chloride of methyl is a liquidised gas. It is manufactured in France when making beet sugar, during a certain stage of the process. I have applied it to sciatic neuritis many times with success. Chloride of methyl must not be confounded with tubes of chloride of ethyl, $C_2$. $H_5$. Ch. which are found in the market. I brought over from France, twelve years ago, a copper flask containing chloride of methyl, which I had McKesson & Robbins refill by sending it back to France, but since then they have kept the chloride of methyl on hand, and I have been able to get my flask filled in New York by them.

Dr. WM. C. KRAUSS, of Buffalo: I am surprised that the doctor did not speak of nitroglycerine. There is great virtue in this drug and the administration of nitroglycerine two or three times a day, increasing it to ten minimums, will relieve a case of sciatica in two or three days sometimes. I reported quite a number of cases treated in this manner at the last meeting of the State Medical Society and since then have treated others with the same good results. Chronic sciatica, of course, does not yield as readily as in the acute cases, and supplemental treatment is necessary in these cases.

Dr. H. L. ELSNER, of Syracuse: In the chronic forms of sciatica neuritis we find osmic acid used hypodermatically directly into the nerve of very great value. It is a remedy which has been used for some time by many neurologists, and in numerous cases where other agents have failed it has proven to be a drug of considerable efficiency. Certainly, it cannot be expected that osmic acid will relieve all symptoms of sciatic neuritis, but in my hands it has relieved a sufficient number of cases to merit further trial. George Jacobi, of New York, a number of years ago, wrote a very excellent paper on the use of osmic acid in these cases, to which I would refer those who are interested in this subject. I have also reported a number of cases in which the drug was used, giving ten or fifteen drops of a one per cent. solution every day or every other day for a few weeks. The solution must be freshly made whenever needed, as it is very likely to undergo change which makes it less efficient.

Dr. R. W. BAMBER, of Kendall: I have found in a few cases, that the application of a spray of ethyl chloride sufficient to freeze the tissues over the tender points had effected a cure after one, two or three applications, when other methods of treatment had failed. The ethyl chloride for this purpose comes in convenient little glass tubes marked "ethyl chloride (Bengue)."

Dr. Angell: In summing up this discussion, I will only call attention to the point of my paper, which was rather intended to emphasise the nature of sciatica than to elaborate its treatment.

Unquestionably various modes of treatment will be efficacious, but a clear understanding of the nature of sciatica is of value in its rational management.

(9) Erythromelalgia. By Henry L. Elsner, M. D., Syracuse; discussed by Dr. Higgins, Dr. Nathan Jacobson, Syracuse, Dr. J. P. Creveling, Auburn, and in conclusion by Dr. H. L. Elsner. (10) Expert testimony. By Frederick Sefton, M. D., Auburn. Discussed by Dr. F. F. Dow, Rochester, Dr. D. M. Totman and Dr. Nathan Jacobson, Syracuse, Dr. A. A. Hubbell, Buffalo, and Dr. H. L. Elsner, Syracuse. (11) Experiences in the antiseptic treatment of typhoid fever. By C. H. Richmond, M. D., Livonia Station; discussed by Dr. A. L. Benedict, Buffalo, Dr. W. S. Ely, Rochester, and in conclusion by Dr. C. H. Richmond. (12) Report of a case illustrating the benefit of laparatomy and drainage in desperate cases of purulent peritonitis. By Henry T. Williams, M. D., Rochester; discussed by Dr. N. W. Soble, Rochester. (13) Syphilis of the brain, with report of three cases. By Wm. C. Krauss, M. D., Buffalo. (14) Should opticians practise medicine. By Alvin A. Hubbell, M. D., Buffalo.

The following papers were read by title: Differential diagnosis of certain diseases of the spinal cord, by Floyd S. Crego, M. D., Buffalo. Pistol-shot marks medico-legally considered, by A. L. Hall, M. D., Fair Haven. Herpes facialis, by A. A. Young, M. D., Newark. Some practical aspects of modern medical science, by John O. Palmer, M. D., Auburn. Rational treatment of asthenopia, by Wheelock Rider, M. D., Rochester. Analysis of one hundred and sixty-four cases of diphtheria treated by antitoxin, by Irving M. Snow, M. D., Buffalo.

Dr. Howe, of Erie, offered the following preamble and resolution, which was accepted:

Whereas, Purulent ophthalmia of infancy produces about one-fifth of all the blind in the asylums of this state and elsewhere; and

Whereas, The proportion of these cases can be decidedly lessened by the employment of Credé's method of preventive treatment; therefore

*Resolved*, That this association approves of any legislation which will make this method obligatory in all lying-in asylums and other institutions in this state supported partly or entirely by public funds.

Upon motion, it was also

*Resolved*, That it is the desire of the members of this association that the president appoint a committee to draft a proposition to present to the legislature on the subject of remedial legislation in regard to expert testimony and report at the next meeting.

The president appointed the following committee: Dr. Frederick Sefton, of Cayuga; Dr. W. S. Ely, of Monroe; Dr. Nathan Jacobson and Dr. Henry L. Elsner, of Onondaga, and Dr. A. A. Hubbell, of Erie.

The committee on juvenile insurance appointed at the last meeting rendered a majority report as follows:

After some inquiry and investigation, it has been unable to obtain any specific information that would lead us to believe that the said law of insurance does stimulate or foster crime.

The report was accepted and the committee discharged.

The officers elected for the ensuing year are: President, Dr. E. B. Angell, Rochester; first vice-president, Dr. Wm. C. Krauss, Buffalo; second vice-president, Dr. F. H. Stephenson, Syracuse. The secretary and the treasurer continue in office, their terms not having expired.

Upon motion, a vote of thanks was extended to the president and the secretary for their efforts in making the meeting a successful one.

